# Supplementary material for: Causal Relationship between Aging and Anorexia Nervosa: A White-Matter-Microstructure-Mediated Mendelian Randomization Analysis
Source: Biomedicines. 2024 Aug 16;12(8):1874. doi: 10.3390/biomedicines12081874 (PMC11351342; doi:10.3390/biomedicines12081874)
Supplement: Supplementary file 1 [file biomedicines-12-01874-s001.zip › Supplementary Figure/Figure S8/Leaveoneout plot of MR analysis for TL to ubm-b-1598.pdf]

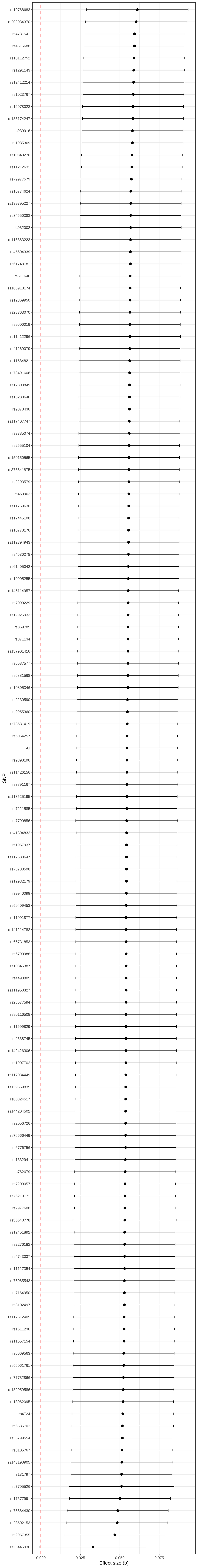

rs10768683
rs202034370
rs4731541
rs4616688
rs10112752
rs1291143
rs12412214
rs1023767
rs16978028
rs185174247
rs939916
rs1985369
rs10840270
rs11212631
rs79977579
rs10774624
rs139795227
rs34550383
rs932002
rs116863223
rs45604339
rs61748181
rs611646
rs188918174
rs12369950
rs28363070
rs9600019
rs11412296
rs41269079
rs11584821
rs78491606
rs17803849
rs13230646
rs9878436
rs117407747
rs3785074
rs2555104
rs150150565
rs376641875
rs2293579
rs450962
rs11769630
rs17445108
rs10773176
rs112394943
rs4530278
rs61405042
rs10905255
rs145114957
rs7099229
rs12925933
rs869785
rs871134
rs137901416
rs6587577
rs6881568
rs10805346
rs2230590
rs9955360
rs73581419
rs6054257
All
rs9398196
rs11426156
rs3891167
rs113525195
rs7221585
rs7790856
rs41304832
rs1957937
rs117630647
rs73730598
rs12932179
rs9940099
rs59409453
rs11991877
rs141214782
rs66731853
rs6790988
rs10845387
rs4498805
rs111950327
rs28577594
rs80116508
rs11699829
rs2538745
rs142426306
rs1907702
rs117034449
rs139669835
rs80324517
rs144204502
rs2056726
rs76666449
rs6776756
rs1332941
rs762679
rs7209057
rs76219171
rs2977608
rs35640778
rs12451892
rs2276182
rs4743037
rs11117354
rs76065543
rs7164950
rs8102497
rs117512405
rs1611236
rs11557154
rs6669563
rs56061761
rs77732866
rs182059586
rs13062095
rs4724
rs6536702
rs56799554
rs8105767
rs143190905
rs131797
rs7705526
rs17677991
rs75664430
rs28502153
rs2967355
rs35446936
SNP
0.000
0.025
0.050
0.075
Effect size (b)
